# A Systematic Review of the Behavioral Responses by Stored-Product Arthropods to Individual or Blends of Microbially Produced Volatile Cues

**DOI:** 10.3390/insects12050391

**Published:** 2021-04-28

**Authors:** Marco A. Ponce, Tania N. Kim, William R. Morrison III

**Affiliations:** 1 Department of Entomology, Kansas State University, 123 W. Waters Hall, 1603 Old Claflin Place, Manhattan, KS 66506, USA; tkim@ksu.edu; 2 USDA, Agricultural Research Service, Center for Grain and Animal Health Research, 1515 College Ave., Manhattan, KS 66502, USA; william.morrison@usda.gov

**Keywords:** post-harvest, chemical ecology, attract-and-kill, semiochemicals, microbially produced volatile organic compounds, integrated pest management, stored products, mycotoxins, fungi

## Abstract

**Simple Summary:**

Microbes are everywhere, including in our food after harvest. Stored-product arthropods have been described as attracted, repelled, or unaffected by microbial cues in the prior published literature, but no one has systematically examined all the prior tests to determine general patterns. We reviewed 43 articles that contained 384 sets of tests on 24 stored-product arthropod species, classifying each response as positive, negative, or net neutral (unaffected or mixed). A total of five and four stored-product arthropods were significantly attracted and repelled by microbial cues, respectively, while 13 were unaffected or exhibited attraction and repellency. We found that the behavioral responses by stored-product arthropods to microbial cues were complex, context-, and species-dependent, which warrants further investigation to clarify mechanisms.

**Abstract:**

Microbes are ubiquitous and play important ecological roles in a variety of habitats. While research has been largely focused on arthropods and microbes separately in the post-harvest supply chain, less attention has been paid to their interactions with each other. Up to this point, there has been no attempt to systematically describe the patterns of behavioral responses by stored-product insects to microbially produced volatile organic compounds (MVOCs). Thus, our aims were to evaluate whether stored-product arthropods were primarily and significantly attracted, repelled, or had a net neutral effect (e.g., unaffected or mixed) by MVOCs presented as (1) complex headspace blends or (2) single constituents and known mixtures. In total, we found 43 articles that contained 384 sets of tests with different combinations of methodology and/or qualitative findings, describing the behavioral responses of 24 stored-product arthropod species from two classes, four orders, and 14 families to 58 individual microbial compounds and the complex headspace blends from at least 78 microbial taxa. A total of five and four stored-product arthropod species were significantly attracted and repelled by MVOCs across odor sources, respectively, while 13 were unaffected or exhibited mixed effects. We summarize the biases in the literature, including that the majority of tests have occurred in the laboratory with a limited subset of methodology and has largely only assessed the preference of adult arthropods. Finally, we identify foundational hypotheses for the roles that MVOCs play for stored-product arthropods as well as gaps in research and future directions, while highlighting that the behavioral responses to MVOCs are complex, context-, and taxon-dependent, which warrants further investigation.

## 1. Introduction

Stored products are essential for the global economy and feeding the planet’s growing population [1]. Each year, the US stores over 16 billion bushels (≈407,264,000 MT) of corn, soybean, and wheat alone [2]. The retail value for just wheat flour in the US is $40 billion [3]. Together, arthropods and microbes represent the two most serious threats to the success of the post-harvest supply chain in delivering nutritious, safe, and sustainable food and food products to consumers and other end users. Globally, losses from both insects and microbes exceed $100 billion annually [4]. While arthropods contribute to governmentally regulated tolerances on insect-damaged kernels and insect fragments, they also result in commodity damage, including contributing to weight loss or downgrades in quality [5]. The presence and spread of microbes in the post-harvest environment is equally undesirable, with potential public health implications [6], and including “off-odor” grain [7], and other deleterious effects on grain quality [8].

Microbes are ubiquitous and play important ecological roles in a variety of habitats. In most environments, microbes are found near the base of the trophic system, and they are responsible for recycling nutrients through a vast “brown web” of decomposers that includes fungi and bacteria [9]. In post-harvest environments, the deterioration of protected commodities that represent a large investment of resources is obviously not desirable. As a result, food facilities adopt a variety of sanitation and drying protocols to preserve the quality of commodities [10]. The moisture in food facilities is generally kept very low, with grain moisture for wheat hovering at about 10% or less to suppress the growth and spread of microbes in the post-harvest environment. However, even when appropriate safeguards are maintained, there may still be issues with “musty” or “off-odor” grain, which has become an increasing complaint by industry. There have been attempts in recent years to classify some these sets of volatiles as biomarkers of commodity quality through a variety of methods [11].

In characterizing the microbial community of grain, community composition is typically a complex assembly of species and is highly variable over time. For example, in warehouse-stored coffee beans, *Saccharomycetales* sp., *Meyerozyma guilliermondii*, and *Pichia kluyveri* dominated in the first six months, but then was dominated by *Wallemia* sp. thereafter [12]. However, the major genera of stored product pathogens include *Alternaria*, *Aspergillus*, *Fusarium*, and *Penicillium* spp., which collectively produced at least 18 different mycotoxins hazardous to human health [13], even in trace amounts [14].

While research has been largely focused on arthropods and microbes separately in the post-harvest supply chain, less attention has been paid to their interactions with each other. However, there is an increasing recognition that insects and microbes jointly contribute to grain quality issues. For example, Ponce et al. [15] found that the presence of microbes increased the fitness of the rice weevil, *Sitophilus oryzae* (L.) (Coleoptera: Curculionidae), at high temperatures, while the presence of both types of pests modified abiotic conditions in ways that were mutually favorable. When the related *Sitophilus zeamais* (Motschulsky) (Coleoptera: Curculionidae) was reared in the presence of the mycotoxin fumonisin B, there was higher progeny production and grain damage than when the mycotoxin was absent [16]. The ham mite, *Tyrophagus putrescentiae* (Schrank) (Acari: Acaridae), was documented vectoring eight fungal species, including *Aspergillus clavatus* and *Penicillium citrinum*, among others [17]. A total of 7–9 stored-product insect taxa in maize stores and in the field were found to have spores belonging to *Aspergillus*, *Fusarium*, *Penicillium* spp., and other fungal taxa on their cuticles [18]. Thus, it appears that stored-product arthropods and microbes may have reciprocal interactions in post-harvest environments.

In addition to their effects on insect population growth and grain quality, microbes may also play important ecological roles by mediating behavioral interactions. For example, microbes are an important source of behaviorally active semiochemicals for insects (reviewed in [19]). Microbial cues may support long-distance habitat finding, short-range host finding, as well as support mate location and reproduction. For example, the microbially produced organic compound (e.g., MVOCs, hereafter), 1-octen-3-ol, synergized attraction to traps with the conspecific aggregation pheromone for *Cryptolestes ferrugineus* Stephens (Coleoptera: Laemophloeidae) [20]. Prior work has demonstrated that the red flour beetle, *Tribolium castaneum* (Herbst) (Coleoptera: Tenebrionidae), used fungi associated with cotton seeds for short-range resource location [21].

Evolutionarily, modern-day stored-product arthropods are descended from species that historically used animal caches of seeds (e.g., [22]) and/or already stressed woody species as food sources (e.g., [23]). It is possible that in the damp environments harboring animal seed caches (e.g., in the ground), fungal cues may have been an indicator of presence of food to stored-product arthropods. Others have even hypothesized that this behavior may result in a genetically conserved response to fungal volatiles by stored-product arthropods [24]. In addition, secondary stored-product arthropods are only able to access nutrients and develop on commodities where the bran has broken or is no longer intact, suggesting that light colonization by fungi may actually allow secondary pests a chance to take advantage of intact kernels that would otherwise be unusable. These life history constraints result is an additional ecological and evolutionary rationale for the attraction of secondary stored-product arthropods to fungal volatiles. Indeed, the red flour beetle, *Tribolium castaneum* (Herbst) (Coleoptera: Tenebrionidae), was found to be attracted to cultures of *Aspergillus niger* and many other fungi [25], suggesting some support for this rationale.

Along with confirmatory studies on the importance of MVOCs to the attraction and orientation by stored-product arthropods, there are published examples suggesting that MVOCs may also have roles as repellents or as neutral stimuli. For instance, though Ahmad et al. [21] found attraction to fungi associated with cotton seeds by *T. castaneum*, Van Winkle et al. [8] found no support for the use of fungal volatiles associated with wheat by the same species. Furthermore, others found 1,315 species oriented to traps baited in agricultural landscapes with the yeast, *Aureobasidium pullulans*, but there were very few oriented to traps with *Penicillium expansum* [26], suggesting that the use of MVOCs is not haphazard by taxa. Moreover, work has found that some MVOCs may act as repellents and thereby disrupt orientation of the granary weevil, *Sitophilus granarius* (L.) (Coleoptera: Curculionidae), to its food source [27]. Each of these suggests that the behavioral response by insects to MVOCs may not be universal to all microbial species and compounds. However, up to this point, there has been no attempt to systematically describe the patterns of behavioral response by stored-product arthropods to MVOCs. Thus, our aims in this study are to evaluate whether stored-product arthropods are primarily attracted, repelled, or unaffected by MVOCs presented as (1) complex headspace blends or (2) single constituents or known component mixtures. We hypothesized that MVOCs may be more important to secondary than primary stored-product arthropods, because they may signal a usable resource to the former but not the latter. In addition, we identify other potential hypotheses for observed patterns, as well as gaps and biases in the literature elucidating MVOC-mediated behavioral changes in stored-product arthropods.

## 2. Materials and Methods

A systematic search of the literature using Google Scholar, (https://scholar.google.com/) and Web of Science (both accessed between 1 May 2020–15 February 2021) were used to identify studies that examined the effects of individual compounds or mixtures of MVOCs on the behavioral responses of stored-product arthropods. Stored-product arthropods were defined as those insects and arachnids attacking stored, durable commodities in the post-harvest supply chain at any of the successive links, including storage, transportation, processing, and marketing. Where applicable, we parsed studies into component experiments where behavioral responses or other factors such as type of assays or measured variables may have differed (e.g., dosage, compound, etc.). We classified each test as resulting in statistically significant attraction (+), repellence (−), or neither (○) compared to a negative or positive control. We excluded any studies lacking appropriate negative or positive controls, lacking replication, or lacking sufficient details on the identity of tested substrates to enable appropriate interpretation. Terms used to search databases included the following singly and/or in combination: “fungal”, “volatiles”, “stored products”, “insect behavior”, “insect–microbe”, “interactions”, “semiochemicals”, “mycotoxin”, “behavioral response”, “attraction”, and “postharvest”, and combinations thereof. In addition, we kept track of the methodology used for tests, response variables, target insect, insect stage, and microbial taxon. We split our analysis up between tests with complex (but usually uncharacterized) blends of MVOCs and those with known individual or known component mixtures of MVOCs. We summarized responses by arthropod species, as well as by microbial cue in order to understand the generalizability of pattern across other categories, and we performed a χ^2^-test in instances with at least three documented tests in the literature to determine whether there were deviations from the null hypothesis of equal frequency of attraction (+), repellency (−), or net neutral responses (○) to MVOCs. Finally, we performed post hoc comparisons to determine whether there were any behavioral differences between the frequency of stored-product arthropods attracted, repelled, or with net neutral responses to individual MVOCs compared to known mixtures of MVOCs. We used R Software for all statistics [28], with α = 0.05. Our search of the literature identified 134 articles that addressed the broad topic of insect–microbe interactions, and 43 articles addressing specifically the behavioral response of stored-product arthropods to MVOCs.

## 3. Results

In total, we found 43 articles (e.g. Supplementary Materials and below) that contained 384 sets of tests with different combinations of methodology and/or qualitative findings (Tables S1 and S2). Overall, the behavioral response of 24 stored-product arthropod species from two classes, four orders, and 14 families to 58 individual microbial compounds and the complex headspace blends from at least 78 microbial taxa was assessed. While studies may have indirectly tested the microbial cues from bacteria in complex headspaces along with fungal cues, no study to date has specifically evaluated the behavioral responses of stored-product arthropods to bacteria alone.

### 3.1. Behavioral Response by Stored-Product Arthropods to Complex Blends of MVOCs

**Figure 1:**
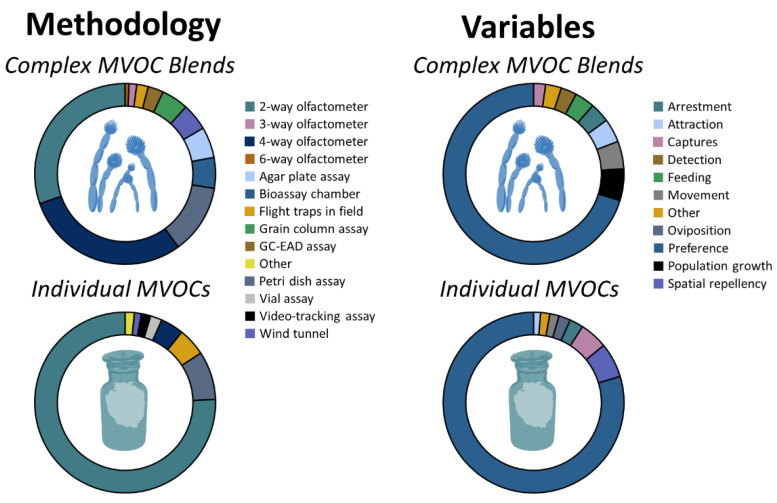
Breakdown of the methodology (**left**) and variables (**right**) used in *n* = 140 tests documenting the behavioral response by stored-product arthropods to complex blends of microbial cues (**top**) and the *n* = 243 tests of individual MVOCs in the primary literature from 43 studies (**bottom**).

In total, there were 140 tests documenting the behavioral response of 16 arthropod taxa to an average of 5 ± 1.6 (mean ± SE) complex blends of microbial cues per taxon (Table S1). The behavioral responses of stored-product arthropods were assessed to complex blends of MVOCs through the combined use of two-way olfactometers, four-way olfactometers, and Petri dish assays in 75% of cases (Figure 1, top-left panel). A total of 91% of the behavioral responses were evaluated in the laboratory. In characterizing behavior, 71% of tests used preference as the primary response variable (Figure 1, top-right panel).

#### 3.1.1. Positive Behavioral Responses

There have been a variety of studies documenting attraction by various combinations of stored-product arthropods to complex blends of microbial cues (Table S1). Overall, complex blends of microbial cues resulted in significant attraction or repellency relative to no response by arthropod taxa (χ^2^ = 5.89; df = 2; *p* < 0.05), with 41% of tests documenting attraction and 34% documenting repellency. In particular, there was significantly more documented attraction across complex blends of microbial cues by three stored-product insect species than expected *a priori*, including by *Carpophilus hemipterus* (Coleoptera: Nitidulidae), *Cryptolestes ferrugineus* (Coleoptera: Laemophloeidae), and *Plodia interpunctella* (Hübner) (Lepidoptera: Pyralidae) (Table 1). Significantly more attraction across stored-product arthropod taxa was exhibited to complex blends of microbial cues emitted from *Alternaria* spp. and *Saccharomyces cerevisiae* (Table 2), both of which are fungi.

#### 3.1.2. Negative Behavioral Responses

There was also a subset of stored-product arthropods that were consistently and significantly repelled by complex microbial cues. A biological control agent, *Lariophagus distinguendus* (Förster) (Hymenoptera: Pteromalidae), and a stored product booklouse, *Liposcelis bostrychophila* Badonnel (Psocoptera: Liposcelididae), were both significantly and consistently repelled by MVOCs (Table 1). Odor sources tested against these species included biodegrading paper, *Eurotium amstelodami* and *Ulocladium botrytis* for the book louse, and two species of *Aspergillus* sp. for the biocontrol agent. Although it was not significantly repelled across all complex MVOCs, the ham mite, *Tyrophagus putrescentiae* (Acari: Acaridae), was repelled by most species of *Aspergillus* sp. (Table S1). By contrast, *Tribolium confusum* du Val (Coleoptera: Tenebrionidae) was both repelled and attracted to many species of *Penicillium*. Since the behavioral responses of stored-product arthropods were often context-dependent, microbial taxon-dependent, and associated with experimental conditions, there were no consistently repellent MVOCs across stored-product arthropod taxa (Table 2).

#### 3.1.3. Net Neutral Behavioral Responses

Overall, in responding to complex blends of MVOCs, 63% of stored-product arthropods had a net neutral behavioral response (e.g., no statistically significant deviation from null). Most of the behavior demonstrated by stored-product arthropods in this category were either nearly equally mixed between one response and another (in 38% of species), or the taxon had low replication of tests in the literature (in 31% of species), with the latter preventing robust statistical evaluation among those subset of cases (Table 1). However, in one case, there was actually significantly more net neutral responses than expected at random, and this was by *T. castaneum*. In particular, the response by *T. castaneum* to complex blends of MVOCs was highly indifferent (Table 1), highlighting its generally ambivalent response to other stimuli. In addition, 80% of the MVOC sources for which there was sufficient replication showed a roughly equal mixture of attraction, repellency, and net neutral responses by stored-product arthropods. The reasons for the large proportion of stored-product arthropods with neutral or mixed responses to MVOCs is discussed further below.

### 3.2. Behavioral Response by Stored-Product Arthropods to Individual or Known Mixtures of MVOCs

**Figure 2:**
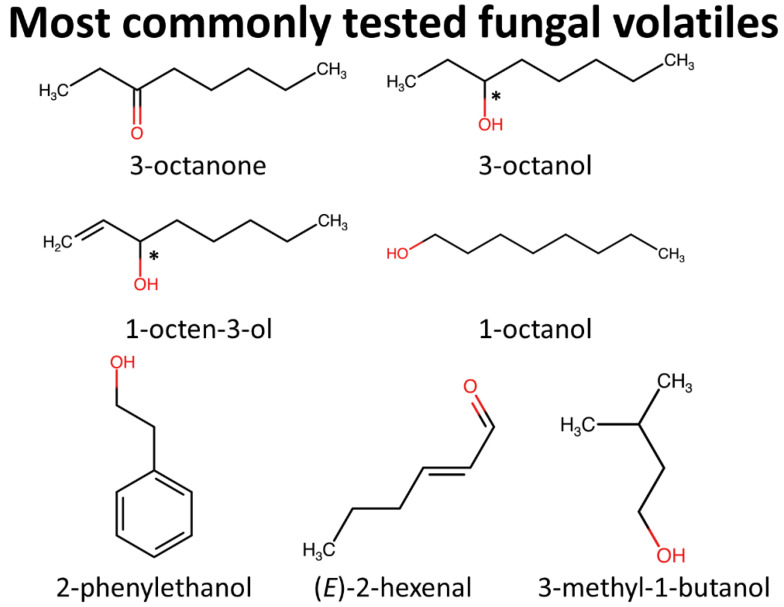
Chemical structures of the most commonly tested MVOCs against stored-product arthropods, which collectively represent 60% of the *n* = 185 tests of individual components in the literature. Functional groups are highlighted in red, while asterisks indicate chiral centers.

In total, there were 243 sets of tests documenting the behavioral response of 19 arthropod taxa to an average of 7 ± 1.5 (mean ± SE) individual microbial compounds per taxon (Table S2; Figure 2). Overall, the responses by stored-product arthropods to 26 individual microbial compounds were evaluated in this study. In particular, the behavioral responses of stored-product arthropods were assessed to individual MVOCs through the use of two-way olfactometers and Petri dish assays in 84% of cases (Figure 1, bottom-left panel), while a total of 93% of tests were performed in the laboratory. In characterizing the behavior of stored-product arthropods, 84% of tests used preference as the primary measure of behavioral response (Figure 1, bottom-right panel).

#### 3.2.1. Positive Behavioral Responses

Some stored-product arthropods had consistently positive responses to individual MVOCs. For example, overall *C. hemipterus*, *T. confusum*, and *T. putrescentiae* significantly preferred or were attracted to individual or known mixtures of microbial compounds (Table 3). Compounds tested against these species included a quaternary mixture of ethyl acetate, acetaldehyde, 2-pentanol, and 3-methyl-1-butanol, as well as an 18-component mixture of known volatiles mimicking *S. cerevisiae*-inoculated banana, and 11 individual microbial compounds (Table S2). When MVOCs were tested individually, there was fairly little consistency in attraction among stored-product arthropods, except in the case of 3-methyl-1-butanol, which elicited more attraction and neutral behavioral responses than repellency (χ^2^ = 22.5; df = 2; *p* < 0.0001; Table 4).

#### 3.2.2. Negative Behavioral Responses

Two stored-product arthropods had negative behavioral responses to MVOCs when tested individually. These included *S. granarius* and *S. zeamais* (Table 3), and they were consistently negative or neutral among 33 individual or known mixtures of MVOCs (Table S2). *Sitophilus zeamais* was repelled by individual MVOCs almost twice as much as *S. granarius*. Overall, among stored-product arthropod taxa, (*E*)-2-hexenal generally elicited repellency significantly more than attraction or neutral responses in about two-thirds of documented cases.

#### 3.2.3. Net Neutral Behavioral Responses

Similar to complex blends, most of the behavior exhibited by stored-product arthropods to individual or known mixtures of MVOCs were either nearly equally mixed between amongst attraction, repellency, and neutrality (in 47% of species; Table 3), or the species had low replication of tests in the literature, as was the case for 26% of species. The latter prevented robust statistical evaluation for those particular species (Table 3). In total, 24 individual MVOCs (or 92% of volatiles with sufficient replication) elicited mixed or multiple responses among stored-product arthropods (Table 4).

#### 3.2.4. Response to Known Mixtures of MVOCs

There were *n* = 22 documented instances of known mixtures of MVOCs tested for the behavioral response of stored-product arthropods (Table S2). The median number of components in each mixture was three MVOCs. Overall, stored-product arthropods were attracted, repelled, or neither to mixtures of MVOCs in 23%, 32%, and 45% of the cases. Stored-product arthropods were significantly more likely to show a net neutral behavioral response (45% in known component mixtures vs. 25% with individual MVOCs; χ^2^ = 5.71; df = 1; *p* < 0.02) but not attraction (23% vs. 37%; χ^2^ = 3.27; df = 1; *p* < 0.07) or repellency (37; χ^2^ = 0.36; df = 1; *p* < 0.55) when MVOCs were presented in mixtures compared to individual MVOCs.

## 4. Discussion

### 4.1. Response by Stored-Product Arthropods to Complex Blends of MVOCs

We found robust evidence for the use of complex blends of MVOCs as stimuli by stored-product arthropod, but roughly equal numbers were repelled or attracted, depending on specific microbial taxon, arthropod species, microbial isolate, and methodology. Interestingly, some stored-product arthropods appear to have fairly idiosyncratic responses to the blends of volatiles from microbes. For example, in one study, *T. confusum* was found to have no preference between isolate 5583 of *Penicillium viridicatum* and whole wheat, but it was found to be significantly repelled by three other isolates of the same fungus (isolates 60, 5572, and 963; [29]). This suggests that other factors might be contributing to the interaction of stored-product arthropods and microbes, such as possibly environmental context, physiological status, priming, or learning.

A common hypothesis in the primary literature is that secondary stored-product arthropods are more likely to respond to MVOCs, since some colonization of a commodity by microbes may be an indicator that broken grain is nearby or that intact kernels may be weakened sufficiently to be used (e.g., [8,24]). This hypothesis appears to be supported with some exceptions, because seven secondary pest species had significant responses to MVOCs compared to three primary pests, and 71% of that subset of secondary pest species responded to MVOCs positively. However, this finding may be a function of research effort in assessing secondary and primary pests in the literature, with 3.5-fold more secondary pests assessed than primary pests assessed in the literature. In the future, the number of species tested should be expanded to include more species with primary pest life histories in stored products.

Interestingly, while many studies have expounded on the importance of MVOCs for *T. castaneum* in particular (e.g., [21,30,31,32]), we found that most (over half) of its documented responses were net neutral. Ambivalence to stimuli by a key stored-product arthropod is an important point, because as several studies have noted [24,32,33,34], most lures perform poorly in robustly attracting *T. castaneum*. While attraction was documented in one-third of cases, the results here suggest that the behavioral response of *T. castaneum* to MVOCs is not indiscriminate, and it may be more complex than previously anticipated. While this pattern of behavioral response does not preclude the use of MVOCs for manipulating the behavior of this important cosmopolitan and damaging insect, it means that more work will need to be undertaken to determine where, to which microbes, and under what conditions *T. castaneum* is attracted to MVOCs. It is possible that *T. castaneum* only responds to a subset of fungi associated with just a handful of commodities.

Overall, we found evidence for significantly fewer net neutral responses to complex blends of MVOCs, with roughly half of documented cases exhibiting attraction by stored-product arthropods and half repellency. At any given time, a grain mass may have multiple microbial species growing, and it may even contain different strains of the same species. In practice, complex microbial species assembly in grain may mean that stored-product arthropods are exposed to very different blends of MVOCs simultaneously. Some of these may have positive and negative effects, and some of these may be antagonistic when binding on the olfactory receptors of arthropods. Antagonistic effects on the behavior of stored-product arthropods appear unavoidable, given the large and diverse assemblage of microbes associated with different commodities [11]. Thus, in the future, it will be important to elucidate how the behavioral responses of stored-product arthropods are altered by headspace with different species richness and/or community composition of microbes. Better elucidating the chemical ecology surrounding microbial–grain–insect interactions will enable an improved understanding of how microbial community assembly affects the behavior of arthropods in postharvest environments.

### 4.2. Response by Stored-Product Arthropods to Individual or Known Mixtures of MVOCs

**Figure 3:**
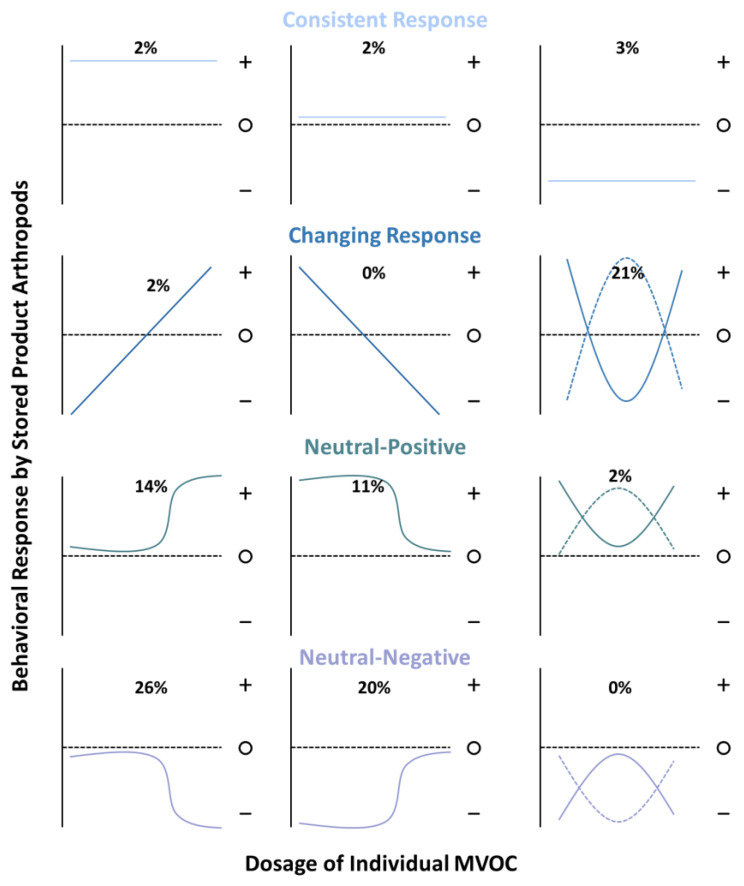
Hypothetical a priori expectations for the behavioral response curves by stored-product arthropods to different dosages of MVOCs and documented responses from published literature. The percentage of cases conforming to each response curve represent a subset (*n* = 66) of total documented cases for which there was information about behavioral response at three or more dosages of an MVOC. The most common groups of responses were the changing response, neutral-positive, and neutral-negative, which together comprised 96% of the cases. Abbreviations: +, significant attraction; ○, neutral response (not statistically different from controls); −, significant repellency.

Many of the individual or mixtures of MVOCs had contrasting effects depending on concentration, often producing a net neutral behavioral effect when aggregated in the tables for this review. However, the dose at which a given MVOC was behaviorally active in manipulating the behavior of stored-product arthropods showed remarkably consistent patterns, with a variety of behavioral response curves possible and somewhat smaller subset documented (e.g., Figure 3). A behavioral response with a significantly negative or positive effect at intermediate dosages was the most and only appreciably frequent among the “changing response” family of curves, comprising 21% of the *n* = 66 cases (e.g., parabolic blue curves). These patterns may arise if moderate mold growth has attractive qualities and MVOCs indicate the presence of mates, usable resources, or preferred microclimate, while it may be repellent if MVOCs indicate overcrowding, compromised resources, or poor oviposition locations. Which one of these is communicated by the MVOC may depend on the particular arthropod, microbial species, prior experience, or physiological status of the individual. Prior work has demonstrated that oviposition by stored-product arthropods generally varies in accordance with the suitability of the substrate [35]. Thus, a possible hypothesis for this response may be that high concentrations of MVOCs represent excess colonization and growth of microbes, which may be inferior oviposition sites, because fungal growth could be detrimental to developing larvae inside kernels.

Neutral-positive behavioral response curves with attraction at high or low dosages comprised a quarter of documented cases (Figure 3; teal lines, left and middle columns). A possible hypothesis for why a secondary pest stored-product arthropod taxon may fall into the neutral-positive grouping with attraction at high but not low dosages of the MVOC may include that a higher concentration is indicative of robust growth by microbes sufficient to compromise the bran of a kernel. An example of this is *Oryzaephilus surinamensis* (Coleoptera: Silvanidae), which was attracted to 1-octen-3-one at high concentrations (0.1–100 μg) but not lower concentrations (0.0001–0.01 μg) [36]. Likewise, *P. interpunctella* was attracted to 1-hexanol at high concentrations [37] but not at lower ones, while the same was also true of *Cryptolestes ferrugineus* and 3-methylbutanol [36]. The MVOCs β-caryophyllene, benzaldehyde, and pentadecane were found to have a neutral–positive effect on *T. putrescentiae*, playing an important role in host location [38]. Prior work has found that the two chemosensory proteins TputCSP1 and TputCPS12 were responsible for recognizing the host volatiles and MVOCs (−)-alloaromadendrene, 2-methylnapthalene, and cyclopentadecane [39].

By contrast, neutral–negative behavioral response curves with repellence at high or low dosages comprised 46% of documented cases, or almost twice the number of neutral–positive cases. These patterns emerged for primary pests such as *S. zeamais*, which may be repelled by high concentrations of fungal volatiles such as (*E*)-2-hexenal and 1-pentanol but have neutral responses when those MVOCs are present in lower concentrations, placing them in the neutral–negative grouping [40]. Overall, the two primary pests, *S. granarius* and *S. zeamais*, exhibited significantly neutral–negative responses to individual MVOCs, including (*E*)-2-hexenal, 1-hexanol, 1-butanol, 1-octanol, 1-pentanal, 1-pentanol, and others [27,40,41,42,43].

There may also be other explanations for the response curves apart from those discussed above (Table 5). For example, there may be innate attraction or repulsion to some compounds [27], learning of habitat cues from the natal environment [44], or physiological constraints in the response of a given taxon to a particular MVOC. In the possible behavioral manipulation of stored-product arthropods by microbes, others have hypothesized that *T. molitor* may be preferentially attracted to infected kernels with *Fusarium*, because it facilitates dispersal of the fungus, and the chemical phenotype in the fungus responsible for attraction was found to be under positive selection [45]. Further studies should determine how widespread possible behavioral manipulation of stored-product arthropods is by microbes found on grain.

### 4.3. Stored-Product Arthropods as Vectors and Ecosystem Engineers for Mycoflora

The post-harvest environment can be thought of as a successional ecosystem [52] and as ecosystem with primary insect colonizers using intact kernels, followed by the colonization of secondary pests that can take advantage of broken kernels and grain dust. Prior work has shown that these species may bring their own suite of microbes, with specific spores associated with the cuticles of each individual [18,52]. For example, the fungal community in bagged coffee was found to shift from being dominated by *Sacharomycetales* during the first six months to dominated by *Wallemia* sp. thereafter [12]. Thus, the microbial community may vary dynamically over time with successive arrivals and dispersal events, which in turn will change the MVOCs emitted by a particular commodity.

Indeed, an increasing amount of research has demonstrated the importance of stored-product arthropods in mediating the mycoflora present in postharvest environments. For example, feeding by insects such as *Sitotroga cerealella* (Olivier) (Lepidoptera: Gelechiidae) on grain kernels was shown to provide a favorable environment for the growth of *F. verticillioides*, as well as promote the production of fumonisin B1 [53,54]. Hotspots of insect infestation in stored grain may significantly elevate the moisture and temperature in the environment, leading to microbial growth and deterioration of the durable commodities [55]. In comparing communities pure or mixed colonies of *S. zeamais* and *Prostephanus truncatus* (Horn) (Coleoptera: Bostrichidae), prior work has found that when both species were present, there was greater microbial growth among the grain [56]. A positive association was found between the survival rate of *R. dominica* and *S. oryzae* in grain and aflatoxin levels [57]. Propagules of *F. proliferatum* and *F. culmorum* were traceable in *T. molitor* feces 20 days after feeding [45]. Even the ham mite, *T. putrescentiae,* was found to disseminate *Fusarium* spp. among stored grain [58].

However, benefits may also accrue to stored-product arthropods by the presence of mycoflora. For example, Ponce et al. [15] found that the presence of both *S. oryzae* and *Aspergillus* spp. in a grain mass altered the abiotic conditions in ways that were favorable to both, and it also resulted in elevated progeny production by *S. oryzae* compared to when microbes were absent. In addition, others have found a positive correlation between insect survival rate and moisture contents of wheat grain [57]. Given the benefits to both microbes and stored-product arthropods by their combined presence and activity in grain, it is clearly reasonable that MVOCs modulate arthropod foraging behavior, even if we have found that it is often taxon-, compound-, and context-dependent.

### 4.4. Biases and Limitations of Reviewed Studies

As we reviewed in the literature, there are some obvious limitations to the available studies. In many of the studies evaluating the behavioral response of stored-product arthropods to complex blends of MVOCs, there is often little or no effort to characterize the composition of those headspace emissions, or if this is done for a microbial taxon, it happens separately from collection of behavioral data. Increasingly, it is important to not just know the behavioral implications of semiochemicals but to have specific information on their identity and how they vary among treatments as well. For example, Van Winkle et al. [8] demonstrated not only attraction by the primary pest, *Rhyzopertha dominica* (F.) (Coleoptera: Bostrichidae) but also went further and described the composition of the MVOCs being tested. Others have also provided similarly extensive information and paired it to behavioral data (e.g., [38]). Pairing behavioral data with GC/MS and other chemical data in the future will help provide a more comprehensive picture of which volatiles may be involved in the behavioral response by stored-product arthropods to certain microbial taxa.

Another significant limitation in the literature is the lack of diversity in behavioral measures of arthropod response to MVOCs. For example, 70–80% of the behavioral responses in all the studies was assessed with preference as a proxy, usually through a two-choice olfactometer, but sometimes with a simple Petri dish assay. However, preference is an imperfect and incomplete measure of behavioral response by stored-product arthropods to MVOCs. Importantly, preference for stimuli is often dependent on the context in which a decision is made by arthropods [59,60], and the “preferred” choice may represent the best of two poor options. As a case in point, in an olfactometer assay, Wright et al. [29] found that *T. confusum* was significantly repelled by 14 isolates of *Pencillium* sp. when choosing against whole wheat but found the same isolates were significantly and highly preferred when choosing against autoclaved wheat. Others have noted that different behavioral assays will result in different conclusions about the same behavioral processes to the same stimuli [61], and thus, using a variety of experimental approaches will help better elucidate how stored-product arthropods use microbial cues. With some exceptions [62,63], there was a notable lack of studies assessing long-distance attraction, distinguishing between arrestment and attraction, and including other important variables such as movement, and whether stored-product arthropods detect many of these microbial cues through GC-EAD. Finally, it is likely that the Petri-dish assays were only testing spatially mediated effects of MVOCs, which is far less useful information when trying to find novel behaviorally relevant semiochemicals for use in traps or management programs. The reason for this is because if a volatile is only active in a small area around where it is located, it is not as useful for manipulating behavior from a distance.

Another severe methodological limitation for >90% of the studies was that only laboratory assays were included. While laboratory studies have their place, including allowing researchers to eliminate extraneous stimuli to determine mechanisms of behavior, it is likewise important to bring the research back into the field to assess whether behavior observed in the laboratory is likely under realistic conditions. Even including assessments under semi-field or semi-natural conditions is helpful in relating laboratory data back to realistic conditions. While some studies assessed microbial cues in traps in the field (e.g., [20]), this could be improved going forward.

We found that individual MVOCs resulted in fewer neutral responses than mixtures. However, this may be an artifact of the low number of mixtures evaluated for just a small subset of species in the literature. A meta-analysis on attraction to lures by herbivores in agroecosystems found that there was greater attraction to mixtures of compounds than individual components [64]. When designing mixtures, it is often important to take into account the relative abundance of compounds in natural headspace in order to evoke similar behavioral responses. In the future, it may be useful to systematically test whether mixtures of MVOCs may be more effective than individual constituents.

Furthermore, while some of the literature reports the ratio or identity of stereoisomer compounds tested, most of the studies did not. Which stereoisomers are used may present an additional source of variation in the data from this review. It is well known that some arthropods, especially moths, may respond to only one specific isomer, or if they respond to multiple isomers, they must be in the correct ratio [65,66]. However, the specific stereoisomer may not be as important for other species [67,68]. Thus, it will be important for future studies to note which specific stereoisomers are used in studies documenting the behavioral response of stored-product arthropods in order to allow for appropriate interpretation.

Finally, with the exception of a handful of studies [69,70,71], the majority of the tests conducted in the literature have been with adult stored-product arthropods. While adults are typically thought of as the dispersing life stage in stored products when designing management strategies [72], grain is readily moved through human-mediated transportation, and even immature secondary pests have a strong dispersal capacity (e.g., *Trogoderma variabile*: [73]), as well as an ability to react differently to volatiles [70]. In the future, other life stages other than adults should be incorporated in tests of behavioral response to MVOCs.

### 4.5. Future Directions in Understanding Response by Stored-Product Arthropods to MVOCs

There have been relatively few studies evaluating how mycotoxins, an important health hazard in the food supply in some parts of the world [14], affect foraging by stored-product arthropods. We have included the few studies that could be found on this in the current study (e.g., [16,47,51,74]), but the lack of breadth in testing of both different mycotoxins and different arthropod species prevents robust generalization. Nonetheless, we do know from these studies that the mycotoxin deoxynivalenol (DON) apparently reduced the locomotory activity of *Tenebrio molitor* as well as induced dose-dependent changes in physiological activity of larvae [74]. Other studies have linked insect activity with increased mycotoxin production in grain [53,57], while some studies have shown that there may be tolerance to mycotoxins [16]. Expanding testing of mycotoxins for how they affect the behavior, development, and growth of stored-product arthropods is a natural next step.

While the studies that have been performed have given us an extensive understanding of some of the preferred MVOCs by stored-product arthropods, future work must expand the characterization of behavior to include other measures. For example, to characterize the behavior of *T. granarium* and *T. variabile*, three behavioral assays were used that included a wind tunnel, olfactometer, and Petri dish assay, which tested attraction, preference, and arrestment, respectively [69]. Combining these tests with others that track the movement of insects (e.g., [75]) and include semi-field and field evaluations of behavior will be helpful in characterizing the full range of behavior elicited by MVOCs as well as understanding response under realistic conditions.

Recently, work has shown that neurogenesis of mushroom bodies in *T. castaneum* is affected by the environment from day four of adulthood and onwards [76]. This has implications for how the olfactory environment may affect the proliferation of neurons, and it is currently unknown how MVOCs in the environment may affect this process. Others have recently made calls to better integrate molecular, genetic, behavior, and chemoecological data to speed applications for semiochemical-mediated, behaviorally-based management for stored-product arthropods [77]. There is little to no mechanistic understanding of how MVOCs encode changes in the behavior or olfaction of stored-product arthropods molecularly, genetically, or in protein expression. At a minimum, using genetic tools such as barcoding will allow for the identification and better characterization of the microbial community on grains to determine which microbial species are most affecting stored-product insect behavior [78]. Taking an integrative approach to the study of MVOCs in the future will be increasingly important to determine new potential targets for RNAi or other novel technology.

## 5. Conclusions

In systematically reviewing the literature on the behavioral responses of stored-product arthropods to complex blends and individual MVOCs, overall, we have found robust support for attraction among complex blends of MVOCs and dose-dependent attraction or repulsion for individual MVOCs that follow typified response patterns. The following stored-product arthropod species were specifically attracted, including *C. hemipterus*, *C. ferrugineus*, *P. interpunctella*, *T. confusum*, and *T. putrescentiae*. By contrast, *L. distinguendus*, *L. bostrychophila*, *S. granarius*, and *S. zeamais* were repelled, while it was much more likely that *T. castaneum* had a net neutral response. Attraction to individual MVOCs by stored-product arthropods was often dose-dependent and compound-specific. The most common behavioral response curves took the form of neutral–positive or neutral–negative, with the latter twice as frequent in the subset of the dataset where multiple dosages had been evaluated. The published literature had some important limitations, including being mostly focused on assessing preference and have been mostly confined to laboratory assays. In the future, the characterization of behavior should include other measures beyond preference such as movement, flight, and field data. Overall, our review has revealed that the effect of MVOCs on the foraging behavior is complex, taxon-, and context-dependent, and it will require more work to tease apart mechanisms driving responses of different species. Future directions include evaluating the behavioral response of stored-product arthropods to mycotoxins; integrating across disciplines to speed research progress; and, expanding the behavioral and biological parameters to characterize the effect of MVOCs.

## Figures and Tables

**Table 1:** Summary of the percent documented occurrences in the primary literature of attraction (+), repellency (−), or no statistical difference from included controls (○) by each of the following stored-product arthropod species among complex blends of microbial cues.

	Behavioral Response						
Arthropod Taxon	−	○	+	χ^2^	*p*	N	Type of Pest ^1^
*Acanthoscelides obtectus*	25	0	75	3.5	0.17	4	1°
***Carpophilus hemipterus***	***0***	***0***	***100***	***10.0***	0.01	5	2°
*Carpophilus humeralis*	0	0	100	-	-	1	2°
***Cryptolestes ferrugineus***	***0***	***14***	***86***	***8.9***	0.01	7	2°
*Cynaeus angustus*	0	0	100	-	-	1	2°
***Lariophagus distinguendus***	***100***	***0***	***0***	***10.0***	0.01	5	wasp
***Liposcelis bostrychophila***	***75***	***25***	***0***	***7.0***	0.03	8	2°
*Oryzaephilus mercator*	0	0	100	-	-	2	2°
*Oryzaephilus surinamensis*	50	0	50	-	-	2	2°
***Plodia interpunctella***	***0***	***0***	***100***	***8.0***	0.02	4	2°
*Sitophilus zeamais*	80	0	20	5.2	0.07	5	1°
*Tenebrio molitor*	43	0	57	3.7	0.16	7	2°
***Tribolium castaneum***	***14***	***55***	***31***	***10.4***	0.01	42	2°
*Tribolium confusum*	46	21	33	3.8	0.15	39	2°
*Typhaea stercorea*	0	0	100	-	-	1	2°
*Tyrophagus putrescentiae*	50	0	50	4.0	0.14	8	2°

Note: arthropods showing a sig. behavioral response bolded and italicized ^1^ Abbreviations: 1°—primary pest, 2°—secondary pest.

**Table 2:** Summary of the percent of documented occurrences in the primary literature of attraction (+), repellency (−), or no statistical difference from included controls (○) across 16 stored-product arthropods to each of the following complex blends of microbial cues.

	Behavioral Response ^1^					
Microbial Taxon^2^	−	○	+	χ^2^	*p*	N
***Alternaria* sp. (extract, plated)**	**0**	**0**	**100**	**6.0**	**0.05**	**3**
*Aspergillus* sp ^3^	48	22	30	2.4	0.30	23
Brewer’s yeast	25	0	75	3.5	0.17	4
Assorted fungal genera ^4^	19	31	50	1.6	0.45	16
Unspecified fungi	14	46	41	3.9	0.14	22
*Fusarium* sp. (culture, plated, extracts, grains inoculated)	35	18	47	2.2	0.33	17
*Penicillum* sp. (pure culture, inoculated grain)	46	20	34	4.4	0.11	41
***Saccharomyces cerevisiae*** **(culture and inoculated food)**	**0**	**0**	**100**	**6.0**	**0.05**	**3**
*Trichoderma harzianum* (multiple strains)	20	20	60	1.6	0.45	5
*Ulocladium botrytis* (two strains, multiple sources)	66	33	0	4.0	0.14	6
**Net behavioral response to microbial cues**	**34**	**24**	**41**	**5.8**	**0.05**	**140**

^1^ Behavioral response by 16 stored-product arthropods, including *Acanthoscelides obtectus*, *Carpophilus hemipterus*, *Carpophilus humerus*, *Cryptolestes ferrugineus*, *Cynaeus angustus*, *Lariophagus distinguendus*, *Liposcelis bostrychophila*, *Oryzaephilus mercator*, *Plodia interpunctella*, *Sitophilus zeamais*, *Tenebrio molitor*, *Tribolium castaneum*, *Tribolium confusum*, *Typhaea stercorea*, and *Tyrophagus putrescentiae*. ^2^ Note: microbes that elicit a consistent and significant behavioral response by stored product arthropods are bolded. ^3^ The genera *Aspergillus*, *Fusarium*, and *Penicillium* have been collapsed into a single row to allow potential generalizations. ^4^ Genera include *Beauveria*, *Byssochamlys*, *Candida*, *Cephalosporium*, *Cladosporum*, *Epicoccum*, *Helminthosporium*, *Hypocrea*, *Microsporum*, *Nigrospora*, *Rhizopous*, *Scopulariopsis*, *Streptomyces*, and *Trichophyton* sp.

**Table 3:** Summary of the percent of documented occurrences in the primary literature of attraction (+), repellency (−), or no statistical difference from included controls (○) by each of the following stored-product arthropod species among individual microbially produced organic compounds.

Arthropod Taxon	Behavioral Response						
	−	○	+	χ^2^	*p*	N	Type of Pest ^1^
*Ahasverus advena*	27	40	33	0.41	0.82	15	2°
*Callosobruchus maculatus*	33	50	17	1.06	0.59	6	1°
***Carpophilus hemipterus^2^***	***0***	***0***	***100***	***6.09***	***0.05***	***3***	***2°***
*Cathartus quadricollis*	40	50	10	2.72	0.26	10	2°
*Cryptolestes ferrugineus*	20	46	34	2.96	0.23	35	2°
*Holepyris sylvanidis*	25	25	50	0.52	0.77	4	wasp
*Lariophagus distinguendus*	50	50	0	-	-	2	wasp
*Oryzaephilus mercator*	20	40	40	1.15	0.56	15	2°
*Oryzaephilus surinamensis*	20	40	40	1.15	0.56	15	2°
*Plodia interpunctella*	13	50	38	1.75	0.42	8	2°
***Sitophilus granarius***	***43***	***45***	***12***	***15.46***	***0.0004***	***69***	***1°***
***Sitophilus zeamais***	***74***	***0***	***26***	***19.7***	***0.0001***	***23***	***1°***
*Tenebrio molitor*	75	25	0	3.55	0.17	4	2°
*Tribolium castaneum*	22	50	28	2.42	0.30	18	2°
***Tribolium confusum***	***0***	***0***	***100***	***8.12***	***0.02***	***4***	***2°***
*Trogoderma granarium*	50	50	0	-	-	2	2°
*Trogoderma inclusum*	50	50	0	-	-	2	2°
*Trogoderma variabile*	100	0	0	-	-	1	2°
***Tyrophagus putrescentiae***	***0***	***0***	***100***	***14.2***	***0.001***	***7***	***2°***
**Net Behavioral Response**	34	37	28	3.8	0.1	243	

^1^ Abbreviations: 1°—primary pest, 2°—secondary pest. ^2^ Note: stored-product arthropods with consistent and significant behavioral response to MVOCS are bolded and italicized.

**Table 4:** The behavioral response among 19 stored-product arthropod species from the primary literature to the following specific microbially produced organic compounds, including documented attraction (+), repellency (−), or no statistical difference from included controls (○).

Microbial Compound ^1^	Behavioral Response					
	−	○	+	χ^2^	*p*	N
***(E)-2-hexenal (with or without wheat)*^2^**	***64***	***7***	***29***	***7.11***	***0.03***	***14*^3^**
(*E*,*E*)-2, 4-decadienal (with or without wheat)	50	0	50	2.06	0.36	4
(*E*,*E*)-2, 4-heptadienal	33	33	33	0.001	1.00	3
(*E*,*E*)-2, 4-nonadienal (with or without wheat)	50	0	50	2.06	0.36	4
1-butanol	25	50	25	0.46	0.80	4
1-octen-3-ol (multiple isomers, with or without maize)	44	33	22	1.35	0.51	18
1-octen-3-one	23	31	46	1.11	0.57	13
1-pentanal	33	33	33	0.001	1.00	3
1-pentanol	50	25	25	0.46	0.80	4
2, 3-butanedione	67	0	33	2.05	0.36	3
2-decanone (with or without wheat)	50	50	0	2.06	0.36	4
2-heptanone (with or without wheat)	50	0	50	2.06	0.36	4
2-hexanone (with or without wheat)	50	0	50	2.06	0.36	4
2-pentanone (with or without wheat)	67	0	33	2.05	0.36	3
2-phenylethanol	31	23	46	1.16	0.56	13
***3-methyl-1-butanol***	***11***	***42***	***47***	***22.5***	***0.0001***	***19***
3-octanone	35	35	29	0.10	0.95	17
butanal (with or without wheat)	50	0	50	2.06	0.36	4
deoxynivalenol	75	0	25	3.58	0.17	4
ethanol	0	50	50	2.06	0.36	6
fumonisin B extract	33	67	0	2.05	0.36	3
heptanal (with or without wheat)	50	0	50	2.06	0.36	4
hexanol (with or without wheat)	50	0	50	2.06	0.36	4
nonanal	0	33	67	2.05	0.36	3
octanol (3-, 1-)	35	29	35	0.16	0.92	17
oleic acid	50	17	33	1.06	0.59	6
***Net Behavioral Response to Compounds***	***37***	***25***	***37***	***6.09***	***0.05***	***185***

^1^ Some compounds may have multiple sources apart from their role as microbial volatiles, including being emitted by grain and other biological sources. ^2^ Note: MVOCs that elicit a consistent and significant behavioral response by stored product arthropods are bolded.^3^ Compounds were left off the table if they had fewer than three documented occurrences in the primary literature.

**Table 5:** Non-exhaustive and non-mutually exclusive hypotheses for the role of MVOCs in driving the behavior of stored-product arthropods described from the primary literature and in the current review.

No.	Short Title	Description	Type of Explanation	Type of Stored Product Pest	Predicted Insect Behavioral Response	Source
1	Host-finding hypothesis	MVOCs used as host-finding kairomones	Eco	1° & 2°	+	[36]
2	Convergent evolution with insects	Similarity in identity of MVOCs to behaviorally active compounds for stored-product arthropods is the result of convergent evolution	Evo	1° & 2°	+	[36]
3	Convergent evolution with grains	Similarity in identity of MVOCs to compounds from headspace of grains is the result of convergent evolution	Evo	n/a	+	[36]
4	Microbe advantage hypothesis	Attractive kairomone-producing microbes may be advantaged by having insects serve as vectors, improve microclimate, feeding on dead insects etc.	Eco/Evo	1° & 2°	+	[16,36,45]
5	Secondary pest benefit hypothesis	MVOCs indicates a resource that may not otherwise be usable to secondary pests because of life history	Eco	2°	+	Multiple sources but see [8,30,46]
6	Symbiotic or non-host hypothesis	Microbes may form symbiotic relationships with stored-product arthropods, or commodity may not be a good host material; MVOCs generally not related to commodity	Eco	1° & 2°	+	[47]
7	Insect advantage hypothesis	Insects may be advantaged by surviving longer, having lower mortality, upregulating metabolic processes, and/or experiencing higher progeny production through as yet determined mechanisms	Eco/Evo	1° & 2°	+	[47,48,49]
8	Mycotoxin tolerance hypothesis	Stored-product arthropods may be able to tolerate the negative consequences of exposure to mycotoxins in order to benefit from other associations with microbes	Eco/Evo	1° & 2°	○	Based on data in [16]
9	Marking pheromone	Some MVOCs similar to insect pheromones might play a role at very low dosages as a marking pheromone to repel conspecifics	Eco/Evo	1° & 2°	−	[36,50]
10	Grain protection hypothesis	MVOCs will generally repel stored-product arthropods, and their production can possibly be induced by grain as a form of protection	Eco/Evo	n/a	−	[51]
11	Primary pest harm hypothesis	MVOCs indicate degraded environment and poor oviposition options for offspring	Evo	1°	−	This contribution
12	Innate response hypothesis	Attraction or repellency to MVOCs is genetically conserved	Evo	1° & 2°	+/−	[27]
13	Learned response hypothesis	Attraction or repellency to MVOCs is learned from natal environment or experience	Eco	1° & 2°, wasps	+/−	[44]

Abbrevations: Eco—ecological rationale or timeframe, Evo—evolutionary, fitness, or genetic rationale, 1°—primary stored-product pests, 2°—secondary stored-product pests.

## Data Availability

The datasets presented in this study can be found in online repositories. The names of the repository/repositories and accession number(s) can be found at: Ponce, Marco A.; Kim, Tania N.; Morrison III, William R. 2021. Data from: A systematic review of the behavioral responses by stored-product arthropods to individual or blends of microbially-produced volatile cues. Ag Data Commons. https://doi.org/10.15482/USDA.ADC/1522297 (accessed on 26 April 2021).

## Supplementary Materials

The following are available online at https://www.mdpi.com/article/10.3390/insects12050391/s1, Table S1: Combinations of stored-product arthropods tested for their behavioral response to an assortment of complex microbial headspaces, including documented occurrences of attraction (+), repellency (−), or no statistical difference from included controls (○) in the primary literature, Table S2: Combinations of stored-product arthropods tested for their behavioral response to an assortment of individual microbial compounds, including documented occurrences of attraction (+), repellency (−), or no statistical difference from included controls (○) in the primary literature. Supplementary references used in the generstion of the dataset in this contribution [79,80,81,82,83,84,85,86,87,88,89,90,91,92,93,94,95,96,97,98,99].

## References

[1] FAO (2018). The State of Food Security and Nutrition in the World.

[2] USDA-NASS (2020). Grain Stocks (January 2020).

[3] USDA-NASS Quickstats v.2.0. https://www.nass.usda.gov/Quick_Stats/.

[4] Wacker F., Adler C., Blank C., Fuerstenau B., Kern P., Mueller-Blenkle C. (2018). Food waste and food losses—Importance of international partnerships and research. Proceedings of the 12th International Working Conference on Stored Product Protection, Berlin, Germany, 7–11 October 2018.

[5] Hagstrum D.W., Subramanyam B. (2006). Fundamentals of Stored-Product Entomology.

[6] Hubert J., Stejskal V., Athanassiou C.G., Throne J.E. (2018). Health hazards associated with arthropod infestation of stored products. Annu. Rev. Entomol..

[7] Schnürer J., Olsson J., Börjesson T. (1999). Fungal volatiles as indicators of food and feeds spoilage. Fungal. Genet. Biol..

[8] Van Winkle T., Ponce M., Quellhorst H., Albin C.E., Bruce A., Kim T.N., Zhu K.Y., Morrison I.W.R. (2021). Microbial volatile organic compounds mediate attraction by a primary but not a secondary stored-product insect pest in wheat. J. Chem. Ecol..

[9] Brabcová V., Štursová M., Baldrian P. (2018). Nutrient content affects the turnover of fungal biomass in forest topsoil and the composition of associated microbial communities. Soil Biol. Biochem..

[10] Morrison III W.R., Bruce A., Wilkins R.V., Albin C., Arthur F.H. (2019). Sanitation improves stored product pest management. Insects.

[11] Tiwari S., Kate A., Mohapatra D., Tripathi M.K., Ray H., Akuli A., Ghosh A., Modhera B. (2020). Volatile organic compounds (VOCs): Biomarkers for quality management of horticultural commodities during storage through e-sensing. Trends Food Sci. Technol..

[12] Broissin-Vargas L.M., Snell-Castro R., Godon J.J., González-Ríos O., Suárez-Quiroz M.L. (2018). Impact of storage conditions on fungal community composition of green coffee beans *Coffea arabica* L. stored in jute sacks during 1 year. J. Appl. Microbiol..

[13] Tripathi A., Alam A., Sharma N., Bhandari A.S. (2021). Mycotoxins, mycotoxicosis, and managing mycotoxin contamination: A review. Bio-management of Postharvest Diseases and Mycotoxigenic Fungi.

[14] Omotayo O.P., Omotayo A.O., Mwanza M., Babalola O.O. (2019). Prevalence of mycotoxins and their consequences on human health. Toxicol. Res..

[15] Ponce M., Kim T.N., Morrison W.R. (2021). The interaction between *Sitophilus oryzae* (Coleoptera: Curculionidae) and Aspergillus flavus jointly affects microclimate, grain quality, and each other’s fitness. Environ. Entomol..

[16] Usseglio V.L., Dambolena J.S., Martinez M.J., Zunino M.P. (2020). The role of fumonisins in the biological interaction between *Fusarium verticillioides* and *Sitophilus zeamais*. J. Chem. Ecol..

[17] Da Silva G.L., Esswein I.Z., Heidrich D., Dresch F., Maciel M.J., Pagani D.M., Valente P., Scroferneker M.L., Johann L., Ferla N.J. (2019). Population growth of the stored product pest *Tyrophagus putrescentiae* (Acari: Acaridae) on environmentally and medically important fungi. Exp. Appl. Acarol..

[18] Lamboni Y., Hell K. (2009). Propagation of mycotoxigenic fungi in maize stores by post-harvest insects. Int. J. Trop. Insect Sci..

[19] Davis T.S., Crippen T.L., Hofstetter R.W., Tomberlin J.K. (2013). Microbial volatile emissions as insect semiochemicals. J. Chem. Ecol..

[20] Stevens M.M., Wood R.M., Mo J. (2019). Monitoring flight activity of *Cryptolestes ferrugineus* (Coleoptera: Laemophloeidae) in outdoor environments using a commercial pheromone lure and the kairomone 1-octen-3-ol. J. Stored Prod. Res..

[21] Ahmad F., Daglish G.J., Ridley A.W., Burrill P.R., Walter G.H. (2013). Short-range resource location by *Tribolium castaneum* Herbst (Coleoptera: Tenebrionidae) demonstrates a strong preference for fungi associated with cotton seed. J. Stored Prod. Res..

[22] Wright V.F., Fleming E.E., Post D., Wright F. (1990). Survival of *Rhyzopertha dominica* (Coleoptera, Bostrichidae) on fruits and seeds collected from woodrat nests in Kansas. J. Kansas Entomol. Soc..

[23] Borgemeister C., Tchabi A., Scholz D. (1998). Trees or stores? The origin of migrating Prostephanus truncatus collected in different ecological habitats in southern Benin. Entomol. Exp. Appl..

[24] Morrison W.R., Scully E.D., Campbell J.F. (2021). Towards developing behaviorally-based integrated pest management programs for stored-product insects. Pest Manag. Sci..

[25] McFarlane D. (2018). The Role of Fungi and Their Associated Volatiles in the Ecology of *Tribolium Castaneum*. Ph.D. Thesis.

[26] Davis T.S., Landolt P.J. (2013). A survey of insect assemblages responding to volatiles from a ubiquitous fungus in an agricultural landscape. J. Chem. Ecol..

[27] Germinara G.S., De Cristofaro A., Rotundo G. (2015). Repellents effectively disrupt the olfactory orientation of *Sitophilus granarius* to wheat kernels. J. Pest Sci. (2004).

[28] Team R.C. R: A language and Environment for Statistical Computing. https://www.r-project.org/.

[29] Wright V.F., Harein P.K., Collins N.A. (1980). Preference of the confused flour beetle for certain Penicillium isolates. Environ. Entomol..

[30] Ahmad F., Daglish G.J., Ridley A.W., Walter G.H. (2012). Responses of *Tribolium castaneum* to olfactory cues from cotton seeds, the fungi associated with cotton seeds, and cereals. Entomol. Exp. Appl..

[31] Seifelnasr Y.E. (1981). Olfactory Response of Red Flour Beetles, *Tribolium castaneum* (Herbst), to Various Forms of Wheat, Millet, and a Fungus as Determined by a Light-Sensitive Apparatus. Master’s Thesis.

[32] Dooley M., Peel A.D., Wakefield M. The responses of *Tribolium castaneum* to wheat germ oil and fungal produced volatiles. Proceedings of the 12th International Working Conference on Stored Product Protection (IWCSPP) in Berlin.

[33] Savoldelli S., Trematerra P. (2011). Mass-trapping, mating-disruption and attracticide methods for managing stored-product insects: Success stories and research needs. Stewart Postharvest Rev..

[34] Campbell J.F. (2012). Attraction of walking *Tribolium castaneum* adults to traps. J. Stored Prod. Res..

[35] Gerken A.R., Campbell J.F. (2020). Oviposition and development of *Tribolium castaneum* Herbst (Coleoptera: Tenebrionidae) on different types of flour. Agronomy.

[36] Pierce A.M., Pierce H.D., Borden J.H., Oehlschlager A.C. (1991). Fungal volatiles: Semiochemicals for stored-product beetles (Coleoptera: Cucujidae). J. Chem. Ecol..

[37] Būda V., Apšegaitė V., Blažytė-Čereškienė L., Butkienė R., Nedveckytė I., Pečiulytė D. (2016). Response of moth *Plodia interpunctella* to volatiles of fungus-infected and uninfected wheat grain. J. Stored Prod. Res..

[38] Li H.P., Yang W.J., Qu S.X., Pei F., Luo X., Mariga A.M., Ma L. (2018). Variation of volatile terpenes in the edible fungi mycelia *Flammulina velutipes* and communications in fungus-mite interactions. Food Res. Int..

[39] Qu S.X., Ma L., Li H.P., Song J.D., Hong X.Y. (2016). Chemosensory proteins involved in host recognition in the stored-food mite *Tyrophagus putrescentiae*. Pest Manag. Sci..

[40] Zunino M.P., Herrera J.M., Pizzolitto R.P., Rubinstein H.R., Zygadlo J.A., Dambolena J.S. (2015). Effect of selected volatiles on two stored pests: The fungus *Fusarium verticillioides* and the maize weevil *Sithophilus zeamais*. J. Agric. Food Chem..

[41] Germinara G.S., De Cristofaro A., Rotundo G. (2008). Behavioral responses of adult *Sitophilus granarius* to individual cereal volatiles. J. Chem. Ecol..

[42] Niewiada A., Nawrot J., Szafranek J., Szafranek B., Synak E., Jeleń H., Wa̧sowicz E. (2005). Some factors affecting egg-laying of the granary weevil (*Sitophilus granarius* L.). J. Stored Prod. Res..

[43] Herrera J.M., Pizzolitto R.P., Zunino M.P., Dambolena S., Zygadlo J.A. (2015). Effect of fungal volatile organic compounds on a fungus and an insect that damage stored maize. J. Stored Prod. Res..

[44] Mbata G.N., Shu S., Phillips T.W., Ramaswamy S.B. (2004). Semiochemical cues used by Pteromalus cerealellae (Hymenoptera: Pteromalidae) to locate its host, *Callosobruchus maculatus* (Coleoptera: Bruchidae). Ann. Entomol. Soc. Am..

[45] Guo Z., Pfohl K., Karlovsky P., Dehne H.W., Altincicek B. (2018). Dissemination of Fusarium proliferatum by mealworm beetle *Tenebrio molitor*. PLoS ONE.

[46] Schulthess F., Cardwell K.F., Gounou S. (2002). The effect of endophytic *Fusarium verticillioides* on infestation of two maize varieties by lepidopterous stemborers and coleopteran grain feeders. Phytopathology.

[47] Blackmer J.L., Phelan P.L. (1991). Effect of physiological state and fungal inoculation on chemically modulated host-plant finding by *Carpophilus hemipterus* and *Carpophilus lugubris*. Entomol. Exp. Appl..

[48] Averill A.L., Prokopy R.J. (1988). Factors influencing release of host-marking pheromone by *Rhagoletis pomonella* flies. J. Chem. Ecol..

[49] Usseglio V.L., Pizzolitto R.P., Rodriguez C., Zunino M.P., Zygadlo J.A., Areco V.A., Dambolena J.S. (2017). Volatile organic compounds from the interaction between *Fusarium verticillioides* and maize kernels as a natural repellents of *Sitophilus zeamais*. J. Stored Prod. Res..

[50] Dunkel F.V. (1988). The relationship of insects to the deterioration of stored grain by fungi. Int. J. Food Microbiol..

[51] Cao A., Santiago R., Ramos A.J., Marín S., Reid L.M., Butrón A. (2013). Environmental factors related to fungal infection and fumonisin accumulation during the development and drying of white maize kernels. Int. J. Food Microbiol..

[52] Fandohan P., Hell K., Marasas W.F.O., Wingfield M.J. (2003). Infection of maize by *Fusarium* species and contamination with fumonisin in Africa. African J. Biotechnol..

[53] Sinha R.P. (1961). Food in India: An Analysis of the Prospects for Self-Sufficiency by 1975-76.

[54] Quellhorst H.E., Athanassiou C.G., Bruce A., Scully E.D., Morrison I.W.R. (2020). Temperature-mediated competition between the invasive larger grain borer (Coleoptera: Bostrichidae) and the cosmopolitan maize weevil (Coleoptera: Curculionidae). Environ. Entomol..

[55] Khan T., Shahid A.A., Khan H.A.A. (2016). Could biorational insecticides be used in the management of aflatoxigenic *Aspergillus parasiticus* and its insect vectors in stored wheat?. PeerJ.

[56] Nesvorná M., Gabrielová L., Hubert J. (2012). Suitability of a range of *Fusarium* species to sustain populations of three stored product mite species (Acari: Astigmata). J. Stored Prod. Res..

[57] Webster B., Bruce T., Pickett J., Hardie J. (2010). Volatiles functioning as host cues in a blend become nonhost cues when presented alone to the black bean aphid. Anim. Behav..

[58] Bruce T.J.A., Pickett J.A. (2011). Perception of plant volatile blends by herbivorous insects—Finding the right mix. Phytochemistry.

[59] Gerken A.R., Scully E.D., Campbell J.F. (2018). Red flour beetle (Coleoptera: Tenebrionidae) response to volatile cues varies with strain and behavioral assay. Environ. Entomol..

[60] Balakrishnan K., Holighaus G., Weißbecker B., Schütz S. (2017). Electroantennographic responses of red flour beetle *Tribolium castaneum* Herbst (Coleoptera: Tenebrionidae) to volatile organic compounds. J. Appl. Entomol..

[61] Balakrishnan K. (2019). Olfactory Responses of Two Coleopteran Species: The Stored Product Pest *Tribolium Castaneum* and the Forest Pest Predator Dastarcus Helophoroides. Ph.D. Thesis.

[62] Szendrei Z., Rodriguez-Saona C. (2010). A meta-analysis of insect pest behavioral manipulation with plant volatiles. Entomol. Exp. Appl..

[63] Athanassiou C., Bray D.P., Hall D.R., Phillips C., Vassilakos T.N. (2018). Factors affecting field performance of pheromone traps for tobacco beetle, *Lasioderma serricorne*, and tobacco moth, *Ephestia elutella*. J. Pest Sci. (2004).

[64] Sammani A.M.P., Dissanayaka D.M.S.K., Wijayaratne L.K.W., Morrison W.R. (2020). Effect of pheromone blend components, sex ratio, and population size on the mating of *Cadra cautella* (Lepidoptera: Pyralidae). J. Insect Sci..

[65] Leskey T.C., Khrimian A., Weber D.C., Aldrich J.C., Short B.D., Lee D.-H., Morrison W.R. (2015). Behavioral responses of the invasive *Halyomorpha halys* (Stål) to traps baited with stereoisomeric mixtures of 10,11-epoxy-1-bisabolen-3-ol. J. Chem. Ecol..

[66] Weber D.C., Morrison W.R., Khrimian A., Rice K.B., Short B.D., Herlihy M.V., Leskey T.C., Nielsen A. (2020). Attractiveness of pheromone components with and without the synergist, methyl (2E,4E,6Z)-2,4,6-decatrienoate, to brown marmorated stink bug (Hemiptera: Pentatomidae). J. Econ. Entomol..

[67] Morrison W.R., Grosdidier R.F., Arthur F.H., Myers S.W., Domingue M.J. (2020). Attraction, arrestment, and preference by immature *Trogoderma variabile* and *Trogoderma granarium* to food and pheromonal stimuli. J. Pest Sci. (2004).

[68] Domingue M.J., Morrison W.R., Yeater K., Myers S.W. (2020). Oleic acid emitted from frozen *Trogoderma* spp. larvae causes conspecific behavioral aversion. Chemoecology.

[69] Guo Z., Döll K., Dastjerdi R., Karlovsky P., Dehne H.W., Altincicek B. (2014). Effect of fungal colonization of wheat grains with *Fusarium* spp. on food choice, weight gain and mortality of meal beetle larvae (*Tenebrio molitor*). PLoS ONE.

[70] Jian F. (2019). Influences of stored-product insect movements on integrated pest management decisions. Insects.

[71] Morrison W.R., Larson N.L., Brabec D., Zhang A. (2019). Methyl benzoate as a putative alternative, environmentally friendly fumigant for the control of stored-product insects. J. Econ. Entomol..

[72] Janković-Tomanić M., Petković B., Todorović D., Vranković J., Perić-Mataruga V. (2019). Physiological and behavioral effects of the mycotoxin deoxynivalenol in *Tenebrio molitor* larvae. J. Stored Prod. Res..

[73] Wilkins R.V., Zhu K.Y., Campbell J.F., Morrison W.R. (2020). Mobility and dispersal of two cosmopolitan stored-product insects are adversely affected by long-lasting insecticide netting in a life stage-dependent manner. J. Econ. Entomol..

[74] Trebels B., Dippel S., Schaaf M., Balakrishnan K., Wimmer E.A., Schachtner J. (2020). Adult neurogenesis in the mushroom bodies of red four beetles (*Tribolium castaneum*, Herbst) is infuenced by the olfactory environment. Sci. Rep..

[75] Maille J., Gerken A., Adrianos S., Arthur F., Campbell J., Oppert B., Perkin L., Scheff D., Morrison I.W., Scully E. (2021). Exploiting chemosensory genomics for improved monitoring and control of stored product pests. Insects.

[76] Begerow D., Nilsson H., Unterseher M., Maier W. (2010). Current state and perspectives of fungal DNA barcoding and rapid identification procedures. Appl. Microbiol. Biotechnol..

[77] Awater S., Fürstenau B. The potential of host-specific volatiles from *Tribolium confusum* larval faeces for luring the ectoparasitoid *Holepyris sylvanidis*. Proceedings of the 12th International Working Conference on Stored Product Protection (IWCSPP).

[78] Bartelt R.J., Wicklow D.T. (1999). Volatiles from *Fusarium verticillioides* (Sacc.) Nirenb. and their attractiveness to nitidulid beetles. J. Agric. Food Chem..

[79] Cui K., He L., Cui G., Zhang T., Chen Y., Zhang T., Mu W., Liu F. (2021). Biological activity of trans-2-hexenal against the storage insect pest *Tribolium castaneum* (Coleoptera: Tenebrionidae) and mycotoxigenic storage fungi. J. Econ. Entomol..

[80] Dolinski M.G., Loschiavo S.R. (1973). The effect of fungi and moisture on the locomotory behavior of the rusty grain beetle, *Cryptolestes ferrugineus* (Coleoptera: Cucujidae). Can. Entomol..

[81] Fürstenau B., Adler C., Schulz H., Hilker M. (2016). Host habitat volatiles enhance the olfactory response of the larval parasitoid *Holepyris sylvanidis* to specifically host-associated cues. Chem. Senses.

[82] Germinara G.S., De Cristofaro A., Rotundo G. (2012). Bioactivity of short-chain aliphatic ketones against adults of the granary weevil, *Sitophilus granarius* (L.). Pest Manag. Sci..

[83] Germinara G.S., Conte A., De Cristofaro A., Lecce L., Di Palma A., Rotundo G., Del Nobile M.A. (2012). Electrophysiological and behavioral activity of (E)-2-hexenal in the granary weevil and its application in food packaging. J. Food Prot..

[84] Green P.W.C. (2005). Substrate selection by *Liposcelis bostrychophila* Badonnel (Psocoptera: Liposcelididae): Effects of insect extracts and biodeteriorated book-paper. J. Stored Prod. Res..

[85] Green P.W.C. (2008). Fungal isolates involved in biodeterioration of book-paper and their effects on substrate selection by *Liposcelis bostrychophila* (Badonnel) (Psocoptera: Liposcelididae). J. Stored Prod. Res..

[86] Kao S.-S., Dunkel F.V., Harein P.K. (1984). Behavioral response of the larger black flour beetle (Coleoptera: Tenebrionidae) to olfactory cues from food sources. J. Econ. Entomol..

[87] Ndomo-Moualeu A.F., Ulrichs C., Adler C. (2016). Behavioral responses of *Callosobruchus maculatus* to volatile organic compounds found in the headspace of dried green pea seeds. J. Pest Sci. (2004).

[88] Phelan P.L., Lin H. (1991). Chemical characterization of fruit and fungal volatiles attractive to dried-fruit beetle, *Carpophilus hemipterus* (L.) (Coleoptera: Nitidulidae). J. Chem. Ecol..

[89] Pierce A.M., Borden J.H., Oehlschlager A.C. (1981). Olfactory response to beetle-produced volatiles and host-food attractants by *Oryzaephilus surinamensis* and *O*. mercator. Can. J. Zool..

[90] Pierce A.M., Borden J.H., Oehlschlager A.C. (1983). Effects of age and population density on response to beetle and food volatiles by *Oryzaephilus surinamensis* and *O. mercator* (Coleoptera: Cucujidae). Environ. Entomol..

[91] Pierce A.M., Pierce H.D., Oehlschlager A.C., Borden J.H. (1990). Attraction of *Oryzaephilus surinamensis* (L.) and *Oryzaephilus mercator* (Fauvel) (Coleoptera: Cucujidae) to some common volatiles of food. J. Chem. Ecol..

[92] Pierce A.M., Pierce H.D., Oehlschlager A.C., Borden J.H. (1991). 1-Octen-3-ol, attractive semiochemical for foreign grain beetle, *Ahasverus advena* (Waltl) (Coleoptera: Cucujidae). J. Chem. Ecol..

[93] Rodríguez-González Á., Casquero P.A., Suárez-Villanueva V., Carro-Huerga G., Álvarez-García S., Mayo-Prieto S., Lorenzana A., Cardoza R.E., Gutiérrez S. (2018). Effect of trichodiene production by *Trichoderma harzianum* on *Acanthoscelides obtectus*. J. Stored Prod. Res..

[94] Starratt A.N., Loschiavo S.R. (1971). Aggregation of the confused flour beetle, *Tribolium confusum*, elicited by mycelial constituents of the fungus *Nigrospora sphaerica*. J. Insect Physiol..

[95] Starratt A.N., Loschiavo S.R. (1972). Aggregation of the confused flour beetle, *Tribolium confusum* (Coleoptera: Tenebrionidae) elicited by fungal triglycerides. Can. Entomol..

[96] Steiner S., Erdmann D., Steidle J.L.M., Ruther J. (2007). Host habitat assessment by a parasitoid using fungal volatiles. Front. Zool..

[97] Tsai W.T., Mason L.J., Woloshuk C.P. (2007). Effect of three stored-grain fungi on the development of *Typhaea stercorea*. J. Stored Prod. Res..

[98] Vanhaelen M., Vanhaelen-Fastré R., Geeraerts J., Wirthlin T. (1978). Cis- and trans-octa-1,5-dien-3-ol, new attractants to the cheese mite *Tyrophagus putrescentiae* (Schrank) (Acarina, Acaridae) identified in *Trichothecium roseum* (Fungi imperfecti). Microbios.

[99] Vanhaelen M., Vanhaelen-Fastré R., Geeraerts J. (1980). Occurrence in mushrooms (Homobasidiomycetes) of cis- and trans-octa-1,5-dien-3-ol, attractants to the cheese mite *Tyrophagus putrescentiae* (Schrank) (Acarina, Acaridae). Experientia.

