# Supplementary material for: A Systematic Review of the Behavioral Responses by Stored-Product Arthropods to Individual or Blends of Microbially Produced Volatile Cues
Source: Insects. 2021 Apr 28;12(5):391. doi: 10.3390/insects12050391 (PMC8145595; doi:10.3390/insects12050391)
Supplement: Supplementary file 1 [file insects-12-00391-s001.zip › insects-1187596-supplementary.pdf]

Supplementary Data

Table S1. Combinations of stored-product arthropods tested for their behavioral response to an assortment of complex microbial headspaces, including documented occurrences of attraction (+), repellency (−), or no statistical difference from included controls (○) in the primary literature.

	% Behavioral Response			
**Arthropod & Microbial Taxon**	**−**	**○**	**+**	**N**
Acanthoscelides obtectus				
Trichoderma harzianum (multiple strains)	25	0	75	4
Carpophilus hemipterus				
Candida krusei (inoculated food)	0	0	100	1
Saccharomyces cerevisiae (pure culture & inoculated food)	0	0	100	3
Carpophilus humeralis				
Fusarium verticillioides (NRRL25457)	0	0	100	1
Cryptolestes ferrugineus				
Aspergillus repens (inoculated grain)	0	0	100	1
Cephalosporium acremonium (inoculated grain)	0	0	100	1
Fusarium sp. (inoculated grain)	0	0	100	1
Penicillium corymbiferum (inoculated grain)	0	0	100	1
Scopulariopsis brevicaulis (inoculated grain)	0	0	100	1
Spoiled grain from field	0	0	100	1
Streptomyces sp. (inoculated grain)	0	100	0	1
Cynaeus angustus				
Cladosporum sp. (inoculated maize flour)	0	0	100	1
Lariophagus distinguendus				
Aspergillus sydowii (inoculated grain)	100	0	0	2
Aspergillus versicolor (inoculated grain)	100	0	0	3
Liposcelis bostrychophila				
Biodeteriorated paper incubated for 8 days	100	0	0	1
Eurotium amstelodami (on malt-agar)	100	0	0	1
Ulocladium botrytis (two strains on paper or agar)	66	33	0	6
Oryzaephilus mercator				
Brewer's yeast	0	0	100	2
Oryzaephilus surinamensis				
Brewer's yeast	50	0	50	2
Plodia interpunctella				
Alternaria alternata	0	0	100	1
Aspergillus flavus	0	0	100	1
Fusarium gramminearum	0	0	100	1

Penicilium aunrantiogriseum	0	0	100	1
Sitophilus zeamais				
Fusarium verticillioides (various forms)	80	0	20	5
Tenebrio molitor				
Beauveria bassiana	100	0	0	1
Fusarium avenaceum	100	0	0	1
Fusarium culmorum	0	0	100	1
Fusarium poae	0	0	100	1
Fusarium proliferatum (pure & inoculated grains)	0	0	100	2
Fusarium sp.	100	0	0	1
Tribolium castaneum				
Alternaria solani (triglycerides extract)	0	0	100	1
Aspergillus glaucus (extracts) with spores	0	0	100	2
Aspergillus niger	0	100	0	1
Aspergillus ochraceus	50	50	0	2
Aspergillus tamarii	0	50	50	2
Aspergillus tubingensis	50	50	0	2
Aspergillus wentii	0	100	0	1
Byssochamlys spectabilis	0	100	0	1
Delinted cotton	50	50	0	2
Epicoccum nigrum	1	0	0	
Fungal isolate (A, B, C, D, E isolate)	20	60	20	5
Fungal isolate (A, B, C, D, E isolate) + wheat seed	0	100	0	5
Fungal-infected wheat kernels (direct & extracts)	0	0	100	3
Fusarium chlamydosporum	0	100	0	1
Fusarium proliferatum	0	100	0	1
Fusarium sp.	0	100	0	1
Helminthosporium sativum (triglycerides extract)	0	0	100	1
Hypocrea sp. (inoculated grain)	0	100	0	1
Linted cotton	0	33	66	3
Nigrospora sphaerica (triglycerides extract)	0	0	100	1
Penicillium citrinum	100	0	0	1
Rhizopus oryzae	0	100	0	2
Trichoderma harzianum	0	100	0	1
Unknown fungi; obtained from cotton	0	0	100	1
Tribolium cofusum				
Fungal-produced acids	0	0	100	1
Penicillium chrysogenum	50	0	50	2
Penicillium citrinum	50	0	50	2
Penicillium crustosum	50	50	0	2

Penicillium cyclopium	50	0	50	2
Penicillium expansum	50	0	50	2
Penicillium griseofulvum	50	50	0	2
Penicillium martensii	50	50	0	2
Penicillium oxalicum (Isolate 787, 2300)	50	25	25	4
Penicillium palitans	50	0	50	2
Penicillium puberulum	50	0	50	2
Penicillium pulvillorum	50	0	50	2
Penicillium rubrum	50	50	0	2
Penicillium stoloniferum	50	0	50	2
Penicillium urticae (patulum)	50	50	0	2
Penicillium viridicatum (Isolate 5572, 5583, 60, 963)	38	25	38	8
Typhaea stercorea				
Aspergillus flavus	0	0	100	1
Tyrophagus putrescentiae				
Alternaria sp. (plated)	0	0	100	1
Aspergillus chevalieri (plated)	0	0	100	1
Aspergillus flavus (plated)	100	0	0	1
Aspergillus fumigatus (plated)	100	0	0	1
Aspergillus niger (plated)	100	0	0	1
Aspergillus oryzae (plated)	100	0	0	1
Microsporum gypseum (plated)	0	0	100	1
Trichophyton mentagrophytes (plated)	0	0	100	1
**Overall response across taxa**	**34**	**24**	**41**	140

Table S2. Combinations of stored-product arthropods tested for their behavioral response to an assortment of individual microbial compounds, including documented occurrences of attraction (+), repellency (−), or no statistical difference from included controls (○) in the primary literature.

	% Behavioral Response			
**Arthropod Taxa & Compound Identity**	**−**	○	+	N
Ahasverus advena				
(R,S)-3-octanol	33	33	33	3
1-octen-3-one	33	33	33	3
2-phenylethanol	50	0	50	2
3-methyl-1-butanol	0	50	50	2
3-octanone	33	33	33	3
ethanol	0	50	50	2
Callosobruchus maculatus	0	0	0	
Binary mixture I [10-5 w/w nonanal, 10-6 w/w (E)-2-octenal]	0	0	100	1
Binary mixture II [10-6 w/w nonanal, 10-6 w/w (E)-2-octenal]	0	0	100	1
Binary mixture III [1-pentanol, 1-octen-3-ol]	0	100	0	1
Quaternary mixture [1-pentanol, nonanal, (E)-2-octenal, 3-carene]	100	0	0	1
Ternary mixture I [1-pentanol, nonanal, (E)-2-octenal]	0	0	100	1
Ternary mixture II [3-carene, nonanal, (E)-2-octenal]	100	0	0	1
Carpophilus hemipterus				
Ethyl acetate, acetaldehyde, 2-pentanol, 3-methyl-1-butanol	0	100	0	1
Synthetic 18-component blend mimicking S. cerevisiae-inoculated banana	0	100	0	2
Cathartus quadricollis	0	0	0	
(R,S)-3-octanol	50	0	50	2
1-octen-3-one	50	0	50	2
2-phenylethanol	50	0	50	2
3-methyl-1-butanol	0	50	50	2
3-octanone	50	0	50	2
Cryptolestes ferrugineus				
(R)-(-)-1-octen-3-ol (multiple isomers, with or without pheromone)	25	50	25	12
(R,S)-3-octanol	33	33	33	3

1-octen-3-one	33	33	33	3
2-ethyl-1-hexanol	0	0	100	1
2-phenylethanol	25	25	50	4
3-methyl-1-butanol	0	33	67	3
3-octanone	25	50	25	4
4-ethylacetophenone	0	0	100	1
hexanoic acid	0	0	100	1
nonanal	0	0	100	1
trans-3-octen-2-one	0	0	100	1
valeraldehyde	0	0	100	1
Holepyris sylvanidis	0	0	0	
(E)-2-nonenal	0	100	0	1
1-pentadecene	0	100	0	1
2-component mixture ((E)-2-nonenal, 1-pentadecene)	100	0	0	1
octanoic acid	0	0	100	1
Lariophagus distinguendus	0	0	0	
1-octen-3-ol	50	0	50	2
Oryzaephilus mercator				
(R,S)-3-octanol	33	33	33	3
1-octen-3-one	0	50	50	2
2-phenylethanol	0	50	50	2
3-methyl-1-butanol	0	50	50	2
3-octanone	33	33	33	3
ethanol	0	50	50	2
propionic acid	100	0	0	1
Oryzaephilus surinamensis				
(R,S)-3-octanol	33	33	33	3
1-octen-3-one	0	50	50	2
2-phenylethanol	33	33	33	3
3-methyl-1-butanol	0	50	50	2
3-octanone	33	33	33	3
ethanol	0	50	50	2
Plodia interpunctella				
1-hexanol	0	50	50	2
3-methyl-1-butanol	33	33	33	3
nonanal	0	50	50	2
Phenylacetalaldehyde	0	0	100	1
Sitophilus granarius				
(E)-2-hexenal (with or without wheat)	33	33	33	3
(E)-2-hexenal + (E,E)-2,4-nonadienal + wheat	50	0	50	2
(E,E)-2,4-decadienal (with or without wheat)	50	0	50	4

(E,E)-2,4-heptadienal	33	33	33	3
(E,E)-2,4-nonadienal (with or without wheat)	50	0	50	4
1-butanol	0	50	50	2
1-hexanol (with or without wheat)	50	0	50	4
1-octanol	0	50	50	2
1-octen-3-ol	0	50	50	2
1-pentanol	33	33	33	3
2,3-butanedione	50	0	50	2
2,3-butanedione + wheat	100	0	0	1
2-heptanone (with or without wheat)	50	0	50	4
2-hexanone (with or without wheat)	50	0	50	4
2- or 3-methyl-1-butanol	0	100	0	2
2-pentanone	50	0	50	2
2-pentanone + wheat	100	0	0	1
butanal (with or without wheat)	50	0	50	4
butanal + (E)-2-hexenal + wheat	50	0	50	2
butanal + (E,E)-2,4-nonadienal + wheat	50	0	50	2
butanal +(E)-2-hexenal + (E,E)-2,4-nonadienal + wheat	50	0	50	2
fufural + wheat	50	0	50	2
heptanal (with or without wheat)	50	0	50	4
hexanal (with or without wheat)	50	0	50	4
Multilayer PCL coating + (E)-2-hexenal packaging	100	0	0	1
pentanal	0	50	50	2
Sitophilus zeamais				
(E)-2-hexenal	100	0	0	1
(E)-2-nonenal	100	0	0	1
(E, Z)-2.6-nonadienal	100	0	0	1
1-butanol	50	50	0	2
1-hexanol	100	0	0	1
1-octen-3-ol	100	0	0	1
1-pentanal	100	0	0	1
1-pentanol	100	0	0	1
2-decanone	50	50	0	2
3-decanone	50	50	0	2
3-methyl-1-butanol	50	50	0	2
3-octanol	100	0	0	1
3-octanone	100	0	0	1
Fumonisin B extract	33	67	0	3
Maize exposed to 1-octen-3-ol	100	0	0	1
Maize exposed to 3-octanol	100	0	0	1
propionic acid	100	0	0	1

Tenebrio molitor				
deoxynivalenol	75	0	25	4
Tribolium castaneum				
1-butanol-3-methyl	0	0	100	1
1-octen-3-ol	100	0	0	1
2,3-butanediol	0	0	100	1
2,3-butanediol + 2-pentanone-4-hydroxy-4-methyl + 1-butanol-3-methyl	0	0	100	2
2-nonanone	0	0	100	1
2-pentanone-4-hydroxy-4-methyl	0	100	0	1
3-methylanisol	0	100	0	1
3-octanone	0	100	0	1
5-methyl-3-heptanone	100	0	0	1
acetic acid	0	0	100	1
acetic acid + 1-butanol-3-methyl	0	0	100	1
butyl acetate	0	100	0	1
hexanal	0	100	0	1
trans-2-hexenal	50	0	50	4
Tribolium confusum				
elaidic acid	0	100	0	1
myristic acid	0	100	0	1
oleic acid	0	100	0	1
stearic acid	0	100	0	1
Trogoderma granarium				
oleic acid	50	0	50	2
Trogoderma inclusum				
oleic acid	50	0	50	2
Trogoderma variabile				
oleic acid	100	0	0	2
Tyrophagus putrescentiae				
benzaldehyde	0	100	0	1
cedrol	0	100	0	1
cis-octa-1,5-dien-3-ol	0	100	0	1
dihydrocarveol	0	100	0	1
trans-octa-1,5-dien-3-ol	0	100	0	1
α-terpilene	0	100	0	1
β-caryophyllene	0	100	0	1
**Net behavioral response**	35	28	37	243
